# Histology of the Oral Mucosa in Patients With BRONJ at III Stage: A Microscopic Study Proves the Unsuitability of Local Mucosal Flaps

**DOI:** 10.4021/jocmr1253e

**Published:** 2013-01-11

**Authors:** Sara Di Lorenzo, Alberto Trapassi, Bartolo Corradino, Adriana Cordova

**Affiliations:** a Dipartimento Di Discipline Chirurgiche Ed Oncologiche, Sezione Chirurgia Plastica , Universita Di Palermo, Italy

**Keywords:** Osteonecrosis of the jaw, Bisphosphonates, Aminobisphosphonates

## Abstract

**Background:**

Bisphosphonate Osteonecrosis of the Jaw (BRONJ) is a newly recognized condition reported in patients treated with aminobisphosphonates (BF). BRONJ is defined as the presence of exposed necrotic alveolar bone that does not resolve over a period of 8 weeks in a patient taking bisphosphonates who has not had radiotherapy to the jaw. Treatment protocols have been outlined, but trials and outcomes of treatment and long-term follow-up data are not yet available. In 2004 an expert panel outlined recommendations for the management of bisphosphonate-associated osteonecrosis of the jaws. Through the histological study of the oral mucosa over the bone necrosis and around the osteonecrosis area in 8 patients affected by BRONJ at III stage, the authors highlight the inappropriateness of the local mucosal flaps to cover the losses of substance of the jaw, BF-related.

**Methods:**

Mucosa tissue was taken from 8 patients, affected by BRONJ, III stage. The samples taken from the mucosa around and over the osteonecrosis area were fixed with formalin and an ematossilina-eosin dichromatic coloring was carried out.

**Results:**

The samples of mucosa showed pathognomonic signs of cell suffering that prove that in these patients using local mucosa flaps is inappropriate.

**Conclusions:**

The authors suggest that only a well vascularized flap as free flap must be used to cover the osteonecrosis area in patients with BRONJ stage III. Because of the structural instability of the mucosa in patients suffering of osteonecrosis Bf related the local flaps are prone to ulceration and to relapse.

## Introduction

Bisphosphonates (BP) are a widely used class of drugs with known efficacy in the prevention and treatment of osteoporosis, Paget’s disease of bone, hypercalcemia of malignancy, osteolytic lesions of multiple myeloma and metastatic osteolytic lesion from breast, lung and other soft tissue tumours [1].

The development of BRONJ lesions appears to be associated with previous dental traumatic injury, like tooth extraction [2, 3], in the majority of cases. Spontaneous cases occur in less than 30% of patients especially localized in areas with very thin overlying mucosa [4]. BRONJ typically occurs in patients who are receiving intravenous BP treatment. However, an increasing frequency of osteonecrosis of the jaw has been reported recently in people who receive oral BPs.

Although numerous studies strongly suggest an association between BP and BRONJ, the true incidence, etiology, pathogenesis, and natural history of this condition have yet to be elucidated.

There are several pathogenic hypothesis about the mechanisms that cause the BRONJ [5]. BPs are thought to function by inhibiting at least one enzyme of the intracellular mevalonate pathway in osteoclasts [5, 6]. Inhibition of this pathway prevents the modification of important signaling proteins, which disrupts osteoclast function and leads to indirect apoptotic cell death. Antiangiogenic and antineoplastic properties have been attributed to BPs.

Most authors emphasize the role of BP in the inhibition of the osteoclastic activity as the drugs that lead to the beginning of osteonecrosis [7]. Bisphosphonate mediated inhibition of osteoclastic function leads to decreased bone resorption and inhibits normal bone turnover. Once incorporated into mineralized bone, BPs, stay in the bone for a long time and have a terminal half-life of many years. As a result, patients who discontinue BP therapy still may be at risk of developing BRONJ several years after they stop taking the drug.

However, this theory of the osteoclast inhibition does not explain the elective localization of the disease in maxillofacial area, supporting instead the hypothesis that other mechanisms determine the BRONJ. In reference to what was stated in literature about oesophageal mucosal and gastric lesions in patients treated with oral BP, some authors underlined that the BP given i.v. have a direct cytotoxic effect on oral mucosal keratinocytes because of a leakage of the drug on the overlying mucosa caused by the dental extractions or by some micro-traumatisms [8]. There is clear documentation of BP toxicity to gastrointestinal epithelia. For example, alendronate and risedronate inhibit cell proliferation in vitro by means of inhibition of farnesil-disphosphonate synthase, the same enzyme which is the target of BP in osteoclasts [8, 9]. The clinical effects of this phenomenon are well recognized in the form of upper gastrointestinal side effects from BP use, and also as ulceration occurring in patients who suck BP tablets [10]. Tooth extraction or other dental trauma result in local release of BP. If the local concentration of drugs is high enough, it inhibits proliferation of adjacent epithelial cells [11] and slows healing of the physical breach in the mucosa, BP uptake into bone is in direct proportion to the local rate of bone turnover and the alveolar ridges have high turnover [12]. This evidence could explain the elective localization in maxilla-facial region. In this anatomic area the alveolar ligament, the periosteum, the gingival mucosa and the dental neck are connected together: this peculiar structure may cause, also without any traumatic events, a contact between BP and the soft tissue [13, 14]. A direct cytotoxic effect on the mucosa epithelia cells could explain the necrosis of the flaps of the oral mucosa harvested to cover the bones in BRONJ stage II and III.

In some reports minimal surgical procedures such as sequestrectomy and coverage of the exposed bone with local mucosal flaps are preferred. The authors agree with the above-mentioned non-invasive procedure of demolition, but not with the use of the local mucosa flaps. In their experience the oral mucosa appears instable, prone to diastase and ulceration. Indeed, this type of reconstruction causes a high incidence of infections and relapses characterized by the bone exposure and let them prefer some other different treatments.

The Authors suggest: sequestrectomy and minimal invasive debridement of the necrotic bone and then reconstruction with microsurgical flaps.

Any procedures must be completed with 0.2% chlorhexidine mouthwash and antibiotic therapy.

Thanks to the histological analysis of the mucosa samples taken 2 cm around the osteonecrosis area and the mucosa over and close to the osteonecrosis, in 8 patients affected by BRONJ stage III, the authors highlighted the histopathological changes that underline the unsuitability of the mucosa flaps to fill in the losses of substance.

## Materials and Methods

Mucosa tissue was taken from 8 patients, affected by BRONJ, III stage, which have been under treatment with BP i.v. (zolendronate) for at least 2 years. They either suffered from bone metastases caused by breast cancer and prostate cancer or from osteolytic lesions as a consequence of Multiple Mieloma.

Histological sampling was made at supralesion and perilesional seat (distance from the necrotic bone 2 cm max). The samples were fixed with formalin and an ematossilina-eosin dichromatic coloring was carried out.

Each preparation was subjected to morphological evaluation by optical microscopy. The taken parameters were: acanthosis, papillomatosis, histological architecture.

## Results

**Figure 1:**
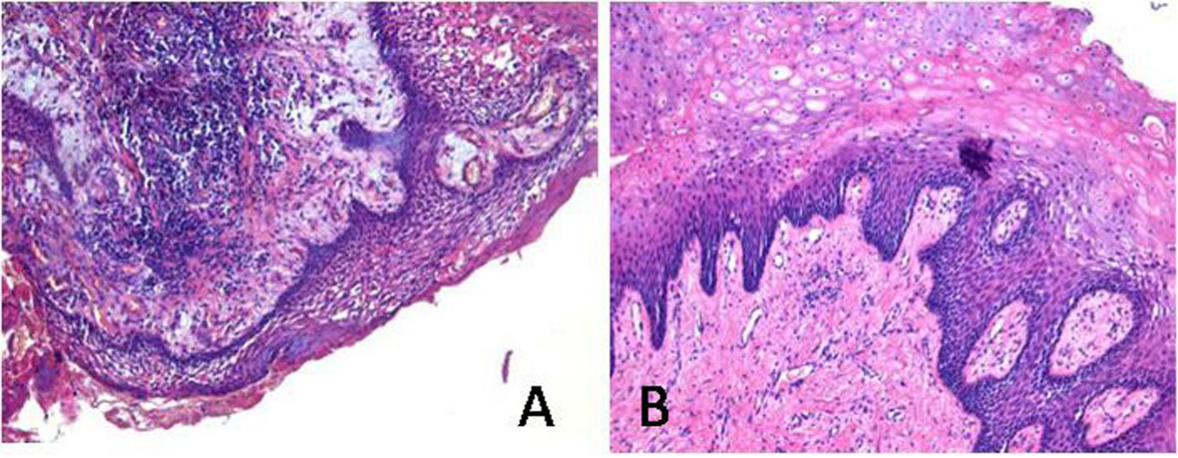
An inflammation of the mucosa with massive presence of plasma cells and granulocytes in the connective tissue was found in the samples taken from above the necrotic bone (A). (B) A conservation of the connective tissue isto-architecture and of deeper epithelial layers was found on the samples of the mucosa surrounding the osteonecrosis. The inflammatory infiltrate is poorly represented and small areas of cellular plasma densification are rarely noticeable. Despite the mucosa deep layers keep their original architecture, the superficial ones are characterized by the presence of swollen and hypereosinophilic cells with a picnotic nucleus, which represents the signs of pathognomonic cell suffering.

An inflammation of the mucosa with massive presence of plasma cells and granulocytes in the connective tissue was found in the samples taken from above the necrotic bone. The histo-architecture of the connective tissue and of the mucosa appears modified: stratification is visible only in some areas and the papillomatosis is clearly noticeable on the slide. The most superficial layers are made up of few mature cells and are characterized by phenomena of ulceration and fray. Also, a phenomenon of interstitial edema is evident. (Fig. 1A).

On the contrary, a conservation of the connective tissue isto-architecture and of deeper epithelial layers was found on the samples of the mucosa surrounding the osteonecrosis. Buds are well defined and regular-shaped. The inflammatory infiltrate is poorly represented and small areas of cellular plasma densification are rarely noticeable. Despite the mucosa deep layers keep their original architecture, the superficial ones are characterized by the presence of swollen and hypereosinophilic cells with a picnotic nucleus, which represents the signs of pathognomonic cell suffering. (Fig. 1B) Thickness has also significantly increased: indeed the granular layer is made up of 16 to 20 orders of cells (usually 2 - 6).

## Discussion

The results of the histopathological examination of the areas affected by BRONJ have shown a reactivity of the tissue to the bone necrosis, and especially a serious damage on the epithelial cells. The clear existence of alterations of the perilesion mucosa (swollen cells, hiperesinophilia, pyknosis) without the typical connective tissue inflammatory infiltration of supra-lesional mucosa, is the evidence that BP cause cellular suffering of the keratinocytes of the oral mucosa. The leakage of BP from the jaw bone, as a consequence of the dental trauma, causes a cell suffering of the surrounding oral mucosa, similar to the pathologies of stomatitis [14], gastritis and esophagitis [15] occurred while administrating BP × os. The process appears to be the same: inhibition of the farnesyl disphosphnate synthase enzyme of the keratinocytes and reduction of the levels of VEGF (vascular endothelium growth factor) due to the action of BP on endothelial cells [5]. As mentioned above, a leak of the drug at soft tissues level adherent to the periosteum, is likely to happen as a result of dental trauma. This event produces a histo-architectural deterioration of the mucosa caused by the contact between BP and basal layers of the same mucosa. Furthermore, due to the features of the parodontal anatomy, the contact between BP and the soft tissues is likely to occur also in the absence of traumatic events, thus justifying the onset of BRONJ even without any dental extraction.

Based on these results, the mucosa surrounding the osteonecrosis area is inert, unstable and prone to ulcerations and infections. Therefore, the coverage of bone substance loss by local mucosa flaps in BRONJ stage III is not recommended, because the arise of diastases of the sutures and septic phenomena are the results of the structural instability and alteration of the mucosal keratinocytes.

Similarly, it is not recommended to use mucosal flaps taken from distant anatomical sites, far from the exposure bone (ex cheek mucosa). Indeed, once the mucosa is transposed on the necrotic bone, as far as it appears healthy and eutrophic, and coming into contact with BP accumulated in the osteonecrosis area, it undergoes the local toxic effect of BP incurring the isto-architectural changes, previously described for the perilesional mucosa.

**Figure 2:**
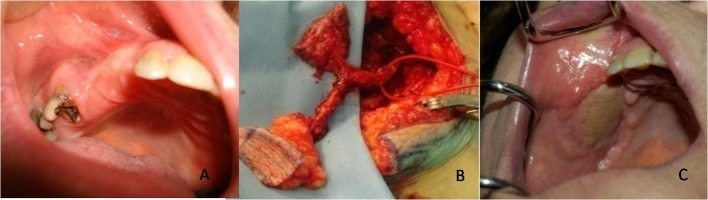
Tha authors suggest the use of microsurgical flap, as a chimeric flaps alt-vastus lateralis muscle to cover the osteonecrosis area of the jaw after a minimal invasive sequestrectomy of the affected bone.

The authors suggest the use of microsurgical flap, as a chimeric flaps alt-vastus lateralis muscle (Fig. 2) to cover the osteonecrosis area of the jaw after a minimal invasive sequestrectomy of the affected bone.
